# Case report: Atypical patterns of nystagmus suggest posterior canal cupulolithiasis and short-arm canalithiasis

**DOI:** 10.3389/fneur.2022.982191

**Published:** 2022-10-10

**Authors:** Janet O. Helminski

**Affiliations:** Department of Physical Therapy, Rosalind Franklin University, North Chicago, IL, United States

**Keywords:** benign paroxysmal positional vertigo, BPPV, posterior canal, downbeat nystagmus, case report, cupulolithiasis, short arm canalithiasis, positional nystagmus

## Abstract

**Background:**

Atypical posterior canal (PC) positional nystagmus may be due to the changes in cupular response dynamics from cupulolithiasis (cu), canalithiasis of the short arm (ca-sa), or a partial/complete obstruction—jam. Factors that change the dynamics are the position of the head in the pitch plane, individual variability in the location of the PC attachment to the utricle and the position of the cupula within the ampulla, and the location of debris within the short arm and on the cupula. The clinical presentation of PC-BPPV-cu is DBN with torsion towards the contralateral side in the DH positions and SHHP or no nystagmus in the ipsilateral DH position and no nystagmus upon return to sitting from each position. The clinical presentation of PC-BPPV-ca-sa is no nystagmus in the DH position and upbeat nystagmus (UBN) with torsion lateralized to the involved side upon return to sitting from each position.

**Case description:**

A 68-year-old woman, diagnosed with BPPV, presented with DBN associated with vertigo in both DH positions and without nystagmus or symptoms on sitting up. In the straight head hanging position (SHHP), the findings of a transient burst of UBN with left torsion associated with vertigo suggested ipsicanal conversion from the left PC-BPPV-cu to canalithiasis. Treatment included a modified canalith repositioning procedure (CRP), which resulted in complete resolution. BPPV recurred 17 days later. Clinical presentation of BPPV included no nystagmus/symptoms in both the contralateral DH position and SHHP, DBN in the ipsilateral DH position without symptoms, and UBN with left torsion associated with severe truncal retropulsion and nausea on sitting up from provoking position. The findings suggested the left PC-BPPV-cu-sa and PC-BPPV-ca-sa. Treatment included neck extension, a modified CRP, and demi-Semont before complete resolution.

**Conclusion:**

An understanding of the biomechanics of the vestibular system is necessary to differentially diagnose atypical PC-BPPV. DH test (DHT) findings suggest that PC-BPPV-cu presents with DBN or no nystagmus in one or two DH positions and sometimes SHHP and without nystagmus or no reversal/reversal of nystagmus on sitting up. The findings suggest PC-BPPV-ca-sa has no nystagmus in DH positions or DBN in the ipsilateral DH position and UBN with torsion lateralized to the involved side on sitting up.

## Introduction

**Figure 1:**
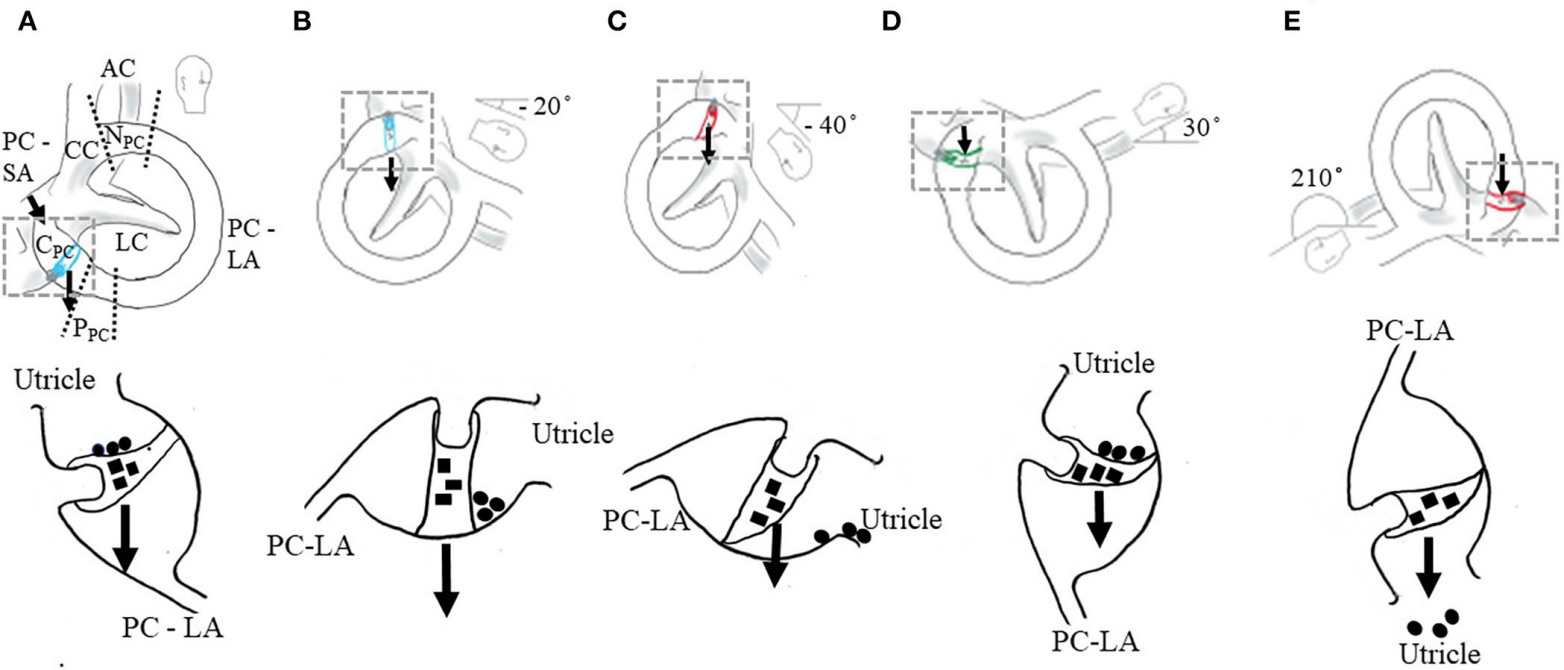
Positional testing (top row) and proposed mechanisms of atypical nystagmus from the PC. The location of debris within canal–cupulolithiasis (■) and canalithiasis of the short arm (•) (bottom row). Illustrated for the right membranous labyrinth. AC, anterior canal; LC, lateral canal; PC, posterior canal; CC, common crus; N_PC_, non-ampullary segment of PC (distal); P_PC_, periampullary segment of PC (proximal); and C_PC_, cupula of PC. The area of the PC between the CC and ampulla is the long arm (LA) and between the ampulla and the utricle is the short arm (SA). The PC long arm has two regions, the periampullary segment—proximal segment near the ampulla and the non-ampullary segment—distal segment near the common crus. Arrow indicates the line of gravity. **(A–C)** Right PC cupulolithiasis. The amount of head extension and flexion in the provoking position will vary the orientation of the axis of the PC cupula relative to the earth horizontal varying the amount and direction of nystagmus. **(A)** Initial position of the Dix-Hallpike test (DHT). In the sitting position, 45° neck rotation toward right (top row). No deflection of cupula. No nystagmus or symptoms (bottom row). **(B)** Head right DH position—recumbent position with the neck rotated 45° toward right and extended 20° below the earth horizontal (top row). Neutral position of ipsilateral PC cupula, the axis of the cupula is oriented in the earth vertical resulting in no nystagmus (bottom row). **(C)** Head right DH position with the neck extended 40° from the earth horizontal (top row). In the pronounced provoking position, the weight of the cupula causes the deflection of the cupula toward the utricle, inhibiting the PC afferent generating a constant, low-amplitude downbeat nystagmus (DBN) with or without torsion lateralized to the uninvolved side (bottom row). **(D,E)** Two earth horizontal positions for the right PC cupula oriented 180° to each other. **(D)** The right half Hallpike (HH) position—recumbent position with the neck rotated 45° toward right and flexed 30° from the earth horizontal. The right PC cupula axis is in the earth horizontal (top row). A weighted cupula causes a maximum deflection of the cupula toward the long arm resulting in the excitation of PC afferent generating an upbeat nystagmus (UBN) with torsion lateralized to the involved side (bottom row). **(E)** Inverted release (IR) position for the right PC cupula. Left side-lying position with the neck flexed 20° and rotated 45° down (top row). A weighted cupula causes a maximum deflection of the cupula toward the utricle, resulting in the inhibition of PC afferent generating a DBN with or without torsion lateralized to the uninvolved side (bottom row).

Benign paroxysmal positional vertigo (BPPV) presents as repeated episodes of positional vertigo that usually last < 1 min. BPPV is the most common cause of vertigo and accounts for 17–42% of all cases of vertigo in adults (1–3). Of all cases of BPPV (*n* = 491), unilateral posterior canal (PC) BPPV and anterior canal (AC) BPPV have the highest and lowest frequency, respectively, accounting for 39 and 4% due to the canal position on the labyrinth (4, 5). BPPV is a mechanical disorder of the inner ear caused by otolithic debris that has separated from the utricular maculae and displaced into the canal–canalithiasis (6) or attached to the cupula–cupulolithiasis (7). With canalithiasis, debris may be located within the short or long arm of the canal (8) (Figure 1A). Debris may be adjacent or not adhere to the cupula in short-arm canalithiasis, changing cupular response dynamics (8–11). Debris may adhere to the cupula on the side of the utricle in cupulolithiasis—short-arm (11) or long-arm cupulolithiasis (10).

The diagnosis of BPPV is based on the history of positional vertigo (12) and the findings on both the Dix-Hallpike test (DHT) (12) and positional maneuvers. The DHT is used to examine the vertical canals. The pattern of nystagmus or no nystagmus in the initial position, in the provoking positions, and on return to the sitting position is crucial to hypothesize the location and direction of movement of the debris within the canal. In the initial sitting position, the clinician rotates the individual's head 45° to the side to be examined, the plane of the PC and AC being vertical. Next, the canal to be tested is rotated within the vertical plane to the provoking position of DHT (DH position). With the head turned 45°, the individual is brought to the supine recumbent position and the head is extended ~20° (12) or 40° (13) below the earth horizontal for a total of ~110–130° of movement in the pitch plane. In the provoking position, with PC-BPPV-ca-la, the otolithic debris settles in the lowest position within the canal, inducing the flow of endolymph in the direction of moving particles and causing nystagmus. If the flow of endolymph moves away from the ampulla (ampullofugal), the afferent is excited and moves towards the ampulla (ampullopetal) the afferent is inhibited. Upon excitation the observed peripheral positional nystagmus is upbeat nystagmus (UBN) towards the forehead) with torsion lateralized to the involved side (the superior pole of both eyes) in the head-referenced coordinate system. When the head is returned to upright, the debris settles toward the ampulla, reversing the flow of endolymph, inhibiting the afferent, and generating a peripheral positional downbeat nystagmus (DBN) (toward the chin) with torsion lateralized to the uninvolved side.

In DH positions and the straight head hanging position (SHHP), the clinician may observe a peripheral DBN, with or without torsion suggesting the activation of one of the vertical canals of the coplanar pair, either AC excitation of the dependent PC inhibition (14, 15). AC excitation is characterized by a DBN with or without torsion lateralized to the involved side that is always observed in the SHHP and sometimes in one or both DH positions due to the orientation of the initial ampullary segment relative to the utricle (16). On sitting up, the inhibition of the AC afferent should generate a reversal of nystagmus. However, there is no reversal of nystagmus or no nystagmus occurs (17). Cambi et al. (14) were the first to suggest that a peripheral and positional DBN may be due to PC inhibition. It was hypothesized that DBN was caused by limited movement of debris to the non-ampullary or distal segment of the PC, referred to as “apogeotropic PC-BPPV” (15, 18), or to the periampullary or proximal segment of the PC, referred to as “sitting up vertigo” (19) due to an obstruction of the PC lumen. “Sitting up vertigo” was associated with or without DBN with torsion lateralized toward the uninvolved side in the DH position or SHHP and UBN with torsion lateralized to the involved side upon return to upright. These obstructions were thought to be due to large fragments of otolithic debris or constriction of the PC lumen (15, 18, 19).

However, emerging evidence suggests that a peripheral DBN observed in the DH position and SHHP may be due to a change in the dynamics of the cupular response because of PC-BPPV cupulolithiasis (PC-BPPV-cu) (8, 9, 20) and PC-BPPV canalithiasis of the short arm (PC-BPPV-ca-sa) (11). It was originally postulated that PC-BPPV-cu, in the ipsilateral DH position (involved side), evoked a gradual-onset, low-amplitude, persistent [>1 min (21)] UBN with torsion toward the involved ear (7, 22). However, the degree of neck extension in the DH position (8, 10), the natural variability of the orientation of the cupula within the population (23, 24), and the location of the debris within the short arm and on the cupula were not considered. These factors influence the patterns of nystagmus and the success of treatment for PC-BPPV-cu (23). In the DH position, if the short arm is connected more superiorly or if the DH position is less pronounced, the head extends ~20° below the earth horizontal, nystagmus may not be observed because the cupula axis is oriented in the earth vertical, and no cupula deflection occurs (Figures 1A,B) (8, 11, 22). In the DH position, if the short arm is connected more inferiorly and/or if the DH position is more pronounced, the head extends ~30–40° below the earth horizontal, the afferent is inhibited and a constant, low-amplitude DBN with or without torsion toward the unaffected side will be observed (Figure 1C) (8, 11) in the contralateral DH position (uninvolved side) and sometimes in the ipsilateral DH position and SHHP (17).

There are no practical clinical guidelines for the management of PC-BPPV-cu, PC-BPPV-ca-sa, and AC-BPPV canalithiasis (12). The purpose of this case report is to describe the clinical presentation, diagnostic process, and particle repositioning maneuvers for an individual who presented with two atypical patterns of positional nystagmus, suggesting a change in PC cupular response dynamics. The first variant was primarily DBN in DH positions with no nystagmus on sitting, suggesting PC-BPPV-cu. Ipsilateral canal conversion from DBN to UBN during the SHHP suggested PC-BPPV-cu on the long-arm side. The second variant was slight DBN in the DH position due to PC-BPPV-cu on the short-arm side and primarily UBN on sitting, suggesting PC-BPPV-ca-sa. This case met all institutional health insurance, portability, and accountability act requirements, and the approval was obtained from Midwestern University's Institute Review Board.

## Patient information

A woman aged 68 years experienced spontaneous episodic vertigo triggered with changes in the head position relative to gravity. She was referred for the management of BPPV by Midwestern University's Multispecialty Clinic and was evaluated 12 days after onset. Neurootologic history included intermittent sensation of pressure, tinnitus, and hyperacoustics of the left ear. She had no significant medical or family history.

## Clinical findings

Clinical neurologic (25) and oculomotor examinations (26) were normal except for mild balance impairment. The clinician observed a slight spontaneous right beat nystagmus in all positions of gaze without fixation. The use of videonystagmography (VNG) (Chartr, Otometrics, Denmark) during positional testing and treatment prevented visual fixation and the suppression of nystagmus. Lab testing, vestibular function testing, and radiographic imaging were not performed. See the timeline for the first and second episodes of care (Table 1).

**Table 1:** Timeline for first and second episode of care including clinical findings, diagnostic assessment, and therapeutic intervention.

**Timeline**	**First variant**		**Second variant**		
	**PC-BPPV-cu-la**		**PC-BPPV-cu-sa and PC-BPPV-ca-sa**		
	**Day 12**	**Day 19**	**Day 32**	**Day 33**	**Day 34**
**Positional testing**					
DHT—head R					
• Direction	• **Persistent DBN**	• None	• None	• Persistent DBN	• None
• Symptoms	• **Vertigo**	• None	• None	• None	• None
Return to upright					
• Direction	• RBN	• None	• **Transient UBN, LT**	• None	• None
• Symptoms	• None	• None	• **Vertigo, truncal retropulsion**	• Imbalance, nausea	• None
DHT—head L					
• Direction	• **Persistent DBN**	• None	• DBN (8 s) to persistent LBN	• Persistent LBN	• None
• Symptoms	• **Vertigo**	• None	• None	• Slight imbalance	• None
Return to upright					
• Direction	• RBN	• None	• **Transient UBN, LT**	• Transient UBN	• None
• Symptoms	• None	• None	• **Vertigo, truncal retropulsion**	• Imbalance, nausea	• None
SHHP					
• Direction	• **Ipsicanal conversion, transient UBN, LT**	• None	• None	• Persistent DBN	• None
• Symptoms	• Vertigo	• None	• None	• None	• None
Return to upright					
• Direction	• Transient DBN, RT	• None	• **Transient UBN, LT**	• None	• None
• Symptoms	• Vertigo	• None	• **Vertigo, truncal retropulsion**	• Slight imbalance	• None
Diagnosis/mechanism	L PC-BPPV-cu-la	BPPV resolved	L PC-BPPV-ca-sa	L PC-BPPV-cu-sa and -ca-sa	BPPV resolved
	Ipsicanal conversion to L PC-BPPV-ca-la				
Therapeutic intervention	• L CRP x3	• Activity restriction	• Neck Extension x3	• Neck Extension x3.	
	• Activity restriction	• Vitamin D levels	• CRP—x1	• CRP—x1	
			• Activity restriction	• Demi-semont x3	
				• Activity restriction	

PC-BPPV, posterior canal benign paroxysmal positional vertigo; ca, canalithiasis; cu, cupulolithiasis; la, long arm; sa, short arm; DBN, downbeat nystagmus; UBN, upbeat nystagmus; T, torsion; R, right; L, left; LBN, left beat nystagmus; RBN, right beat nystagmus; CRP, canalith repositioning procedure. Bold values key findings.

## First variant: Peripheral DBN DH positions

### Diagnostic assessment of the first variant: Peripheral DBN provoking positions

In the first episode of care, clinicians observed in both DH positions with the head positioned ~40° below the earth horizontal (13) a DBN (no torsion) with a short latency of onset, long duration (>1 min), and crescendo–decrescendo velocity profile associated with symptoms of vertigo. On return to upright from both DH positions, the clinician observed a right beat nystagmus and the patient reported no symptoms. In the SHHP, there was a 60 s latency until the onset of a transient burst of UBN with left torsion, associated with an intense sensation of vertigo. On return to sitting, the clinician observed a burst of DBN with right torsion associated with vertigo (Supplementary Video S1).

### Interpretation of the first variant: Peripheral DBN provoking positions

Central positional vertigo is associated with neurologic findings and, in most cases, impaired pursuit (27). In this case, the history and findings of DBN in both DH positions and the absence of associated neurologic signs suggested vertical canal involvement—either the inhibition of PC or the excitation of AC. Without fixation, the patient had a mild spontaneous right-beating nystagmus (RBN) that increased in intensity without symptoms on return to upright from DH positions. The patient had a history of left neurootologic involvement, which might have resulted in a peripheral RBN or had a pseudo-spontaneous nystagmus. The persistence of DBN in DH positions and the absence of nystagmus in the SHHP suggests PC involvement. In the SHHP, DBN is always observed with AC-BPPV and sometimes with PC involvement (17). With canalithiasis of the AC, the duration of attacks is < 1 min (21, 28). Initially, in the SHHP, the clinician did not observe any nystagmus, suggesting PC-BPPV. At 60 s, the transient burst of UBN with left torsion suggested the excitation of primary afferents to the left PC and an ipsicanal switch, conversion of PC-BPPV-cu-la to PC-BPPV-ca-la (8). The nystagmus changed the direction from a DBN in DH positions to an UBN in SHHP. Left torsion with UBN suggested left ear involvement. On sitting up, DBN with right torsion—suggested the inhibition of the left PC afferent associated with PC-BPPV-ca-la. Based on the history and the ipsicanal switch on positional testing, no further vestibular function testing or radiographic imaging was indicated. The patient's initial symptoms were consistent with those of atypical BPPV involving the PC (8). If she did not show any improvement in or the resolution of symptoms after two to three sessions of particle repositioning maneuver, she would be referred for further diagnostic testing to rule out central involvement (12).

### Therapeutic intervention of the first variant: Peripheral DBN provoking positions

Typical left PC-BPPV was treated with three cycles of a modified canalith repositioning procedure (CRP) (29). In the first cycle, an orthotropic UBN with left torsion was observed in the first and second positions, mild DBN in the right side lying position and no positional nystagmus on return to upright. Activity restrictions were provided due to the complexity of BPPV (12) and were consistent with those provided to patients with intractable BPPV (30). Restrictions included sleeping with the head of the bed elevated to 40°, sleeping on the back or right side, and limiting up/down head movements for 1 week (30).

### Follow-up and outcomes of the first variant: Peripheral DBN provoking positions

In a 1-week follow-up, the patient reported no symptoms of vertigo with daily routine. Positional testing was negative, suggesting that left PC-BPPV had resolved. The patient was relieved that BPPV was resolved and the concern was that it would recur. Activity restrictions were continued, and vitamin D levels were reviewed and found to be within the recommended range (31) to minimize the risk of recurrence of BPPV.

## Second variant recurrence: UBN sitting up

### Diagnostic assessment—second variant recurrence—first session UBN sitting up

Approximately 17 days following the resolution of atypical PC-BPPV, the patient rolled over in the bed and experienced the sensation of vertigo. Clinical examination findings 3 days following the recurrence suggested no associated neurologic signs. The primary complaint was imbalance and nausea on sitting up and with walking. She reported falling while ascending a riser without a rail. Positional testing was performed with VNG (without fixation). With the right DH position and SHHP, the clinician observed no nystagmus, and the patient reported no symptoms. With the left DH position, there was a mild DBN for 8 s followed by an LBN and no symptoms. On return to upright from each position, the clinician observed a transient burst of UBN with left torsion associated with a strong sitting up vertigo and truncal retropulsion (Supplementary Video S2).

### Interpretation—recurrence of the second variant—first session UBN sitting up

The findings suggest PC-BPPV-cu-sa and PC-BPPV-ca-sa. Nystagmus or symptoms are absent in the contralateral DH position and SHHP, but there is a mild DBN in the ipsilateral (left) DH position, which suggests left PC-BPPV-cu-sa. Due to the orientation of the axis of the cupula relative to the earth horizontal, an inhibitory response was generated resulting in DBN. In 19% of patients, following successful particle repositioning maneuvers, a DBN in the ipsilateral DH position suggests the movement of otolithic debris from the long arm to the short arm during the maneuver (11). UBN associated with left torsion on return to upright from all positions and without other neurologic signs suggests vertical canal involvement. On sitting up, PC otolith debris moved from the utricle to the cupula, deflecting the cupula away from the utricle causing the excitation of the afferent generating an UBN with associated torsion (11). Left torsion suggests the excitation of the left PC. The findings do not suggest a periampullary jam in the PC lumen (19) [periampullary restricted canalolithiasis model (32)] based on the history of recent successful treatment of the left PC-BPPV and no nystagmus observed in the contralateral DH position. It is proposed that, with a periampullary jam, an increase in DBN with torsional nystagmus is observed in the contralateral DH position and no nystagmus in the ipsilateral DH position (19). In this case, no nystagmus was observed in the contralateral DH position and DBN was observed in the ipsilateral position. In the ipsilateral DH position following DBN, an LBN was observed, which suggests possible debris settlement in the long arm of the LC.

#### Therapeutic intervention—recurrence of the second variant—first session UBN sitting up

Atypical left PC-BPPV was treated with three cycles of neck extension (33) and a modified CRP (29). The patient was provided with post maneuver activity restrictions. Following the maneuver, the patient reported less intense truncal retropulsion and nausea on sitting up and imbalance on walking. Symptoms gradually resolved over 4 h.

### Diagnostic assessment—recurrence of the second variant—second session

The next day, the individual woke up with severe vertigo and imbalance. She was escorted by her husband to the clinic. On examination, the clinician observed a DBN in the contralateral DH position and SHHP and a LBN in the ipsilateral DH position. On return to upright from the contralateral provoking position and SHHP, there was no nystagmus but there was a slight UBN upon return to upright from the ipsilateral provoking position.

### Interpretation—evolution of the second variant—second session

Positional test findings suggest the left PC-BPPV-cu-sa. The debris within the short arm may be adherent—PC cupulolithiasis of the short arm or adjacent to the cupula (nonadherent)—PC-ca-sa (11). An inverted release (IR) position (lying on the uninvolved side with the head flexed 20° and rotated 45° downward) would determine if the debris was attached to the cupula. In case of being adjacent to the cupula, no nystagmus would occur because the debris would move from the cupula to the utricle (10). LBN in the SHHP may be due to a pseudo-spontaneous nystagmus from the left lateral canal.

### Therapeutic intervention—evolution of the second variant—second session

The patient was treated with neck extension (33) in an attempt to create ipsicanal conversion followed by demi-Semont (18), the final position the same as IR, to remove particles from the cupula on the side of the utricle. The patient were provided with post maneuver activity restrictions, and continued to complain of imbalance and nausea. In the evening, BPPV resolved spontaneously.

### Follow-up and outcomes—evolution of the second variant—second session

At a 1-week follow-up, she had no vertigo or imbalance with her daily routine and positional testing was negative. She was grateful to have resolved BPPV but was concerned about its recurrence.

## Discussion

This case report describes the clinical presentation of two variants of atypical nystagmus from PC-BPPV due to a change in cupular response dynamics, one from cupulolithiasis with debris on the long-arm side and the other from primarily short arm canalithiasis and cupulolithiasis. The clinical presentation of the first variant from PC-BPPV-cu-la was persistent DBN with torsion lateralized to the uninvolved side, associated with symptoms of vertigo in DH positions and no nystagmus or associated symptoms on return to upright. An ipsicanal conversion occurred from PC-BPPV-cu with debris on the long-arm side to canalithiasis in the long arm. This occurred when the patterns of nystagmus changed from an atypical persistent positional DBN in the DH position to a typical positional transient UBN with torsion lateralized toward the involved side (60 s delay to onset) in the SHHP. This ipsilateral canal conversion supported the mechanism of the first variant as PC-BPPV-cu-la. PC-BPPV was successfully treated with a modified CRP. BPPV recurred 17 days later. Her primary complaint was imbalance and nausea. The second variant was both PC-BPPV-cu-sa and PC-BPPV-ca-sa. The clinical presentation of PC-BPPV-cu-sa was no nystagmus or symptoms in the contralateral DH position and SHHP, and DBN with torsion lateralized to the uninvolved side (duration of 8 s) followed by an RBN in the ipsilateral DH position. A burst of UBN with torsion to the involved side associated with symptoms of imbalance and nausea on return to upright from all provoking positions suggested PC-BPPV-ca-sa. The patient was treated with neck extension and a modified CRP. Symptoms gradually resolved over 4 h. The next morning, the patient awoke with symptoms of imbalance and nausea. The clinical presentation was DBN with torsion away from the involved side in DH positions and SHHP, suggesting PC-BPPV-cu. There was a subtle UBN with torsion toward the involved side on return to upright from the ipsilateral DH position, suggesting PC-BPPV-ca-sa. She was successfully treated with neck extension and demi-Semont maneuver. Symptoms gradually resolved within 4 h.

Emerging evidence suggests that atypical positional DBN from the PC may be due to the changes in the cupular response from cupulolithiasis or short-arm canalithiasis (8, 11, 32). The following factors will change the orientation of the cupula axis with respect to the earth horizontal, influencing the patterns of nystagmus and the success of treatment, especially in the case of PC-BPPV-cu and PC-BPPV-ca-sa. The ability to position the head in extension may be hindered by cervical or thoracic spine mobility limitations or pain, necessitating modifications to the positions. The examiner may choose to perform a DH position that has less or more pronounced head extension in the supine (Figure 1B) (12) or head extension more pronounced in the supine recumbent position (Figure 1C) (13). If less pronounced, the head is extended ~20° below the earth horizontal, no nystagmus is observed if the axis of the cupula is oriented in the earth vertical and no cupula deflection occurs (Figures 1A,B) (8, 11, 22). If more pronounced, the head extends ~30–40° below the earth horizontal, the afferent is inhibited and a constant, low-amplitude DBN with or without torsion towards the uninvolved side will be observed (Figure 1C) (11, 12). The patterns of nystagmus will also be influenced by the orientation of the cupula due to the natural individual variability in the location of PC attachment to the utricle (23) and the position of the cupula within the ampulla (24). In the DH position, if the short arm is connected more superiorly, nystagmus may not be observed because the axis of the cupula is oriented in the earth vertical and no deflection of the cupula occurs (Figures 1A,B) (8, 11). If the short arm is connected more inferiorly, the afferent is inhibited and a constant, low-amplitude DBN with or without torsion toward the unaffected side will be observed (Figure 1C) (8, 11). Lastly, the location of the debris within the short arm (11), the adherence of debris to the cupula on the side of the utricle or long arm, and the proximity of debris to the cupula (adjacent) (11) will influence the patterns of nystagmus and the particle repositioning maneuver performed to successfully treat PC involvement.

When atypical nystagmus from the PC is suspected, additional positional testing should be performed to identify the location of the debris within the canal. The half Hallpike (HH) position and IR position may be used to differentiate between PC-BPPV-cu and PC-BPPV-ca-sa and to also differentiate PC-BPPV-cu from AC-BPPV canalithiasis (10, 21). The cupula is deflected maximally by the gravitational force when the axis of the affected cupula is oriented in the earth horizontal. Two earth horizontal positions oriented at 180° to each other: the HH (recumbent supine position with the head rotated 45° toward the involved side, flexed 30°) (10, 22) and the IR position (lying on the uninvolved side with the neck flexed 20°, rotated 45° downward) (10) (Figures 1D,E). If the vertical canals are involved, in the HH, the direction of nystagmus is UBN with torsion lateralized to the involved side. With the IR, the direction of nystagmus reverses the direction relative to the HH position. If nystagmus is stronger in the HH position, it is generated by the posterior cupula due to the inhibition of the PC and if stronger in the inverse position it is generated by the contralateral anterior cupula due to the excitation of the PC (10). If no nystagmus is observed in the IR, otolithic debris may be adjacent to the cupula within the short arm of the PC—PC-BPPV-ca-sa. Persistent positional DBN in the ipsilateral DH position following a successful particle repositioning maneuver for PC-BPPV may be due to ipsilateral PC-BPPV-cu-sa (11). As a result of the maneuver, debris is repositioned from the long arm to the utricle, then to the short arm, and finally settles adjacent to the cupula (11).

PC-BPPV-cu and PC-BPPV-ca-sa have also been implicated as a mechanism of type 2 BPPV (11). The proposed diagnostic criteria for type 2 BPPV are symptoms suggestive of BPPV without nystagmus during the DHT or supine roll test and a short episode of vertigo with truncal retropulsion during and immediately after sitting up from the ipsilesional side (34). Recently, Harmat et al. (11) hypothesized two possible mechanisms of type 2 BPPV, PC-BPPV-ca-sa with debris located inferiorly at the base of the PC cupula incapable of deflecting the cupula or PC-BPPV-cu-sa with debris attached to the surface of the cupula on the side of the utricle loading the cupula but not changing its position. This further supports the role of the cupula in atypical PC-BPPV.

It is recognized that atypical nystagmus from the PC may be generated by limited movement of the debris within the lumen caused by a partial or complete obstruction from an innate stenosis of the canal and/or large fragments of utricular otolithic debris in a normal canal (35). The location of the obstruction determines the presentation of atypical PC-BPPV (Figure 1A). Vannucchi et al. (15) suggested that an obstruction of the lumen of the PC located in the distal or non-ampullary segment of the long arm of the PC near the common crus (Figure 1A) would generate a DBN associated with torsion lateralized to the uninvolved ear or without torsion due to the inhibition of the PC afferent. On return to sitting, there would be no nystagmus because the non-ampullary segment would be oriented in the earth horizontal (Figure 1A). This pattern of nystagmus was referred to as “apogeotropic PC-BPPV” (15, 18) and was further described by Cambi et al. (14) and Califano et al. (17). Scocco et al. (19) later suggested that an obstruction may occur in the periampullary segment (proximal segment) of the long arm of the PC (Figure 1) resulting in “sitting up vertigo.” The pattern of nystagmus suggestive of a periampullary obstruction is no nystagmus in the ipsilateral DH position, and it is either no nystagmus or persistent DBN with or without torsion lateralized to the uninvolved side in the contralateral DH or SHHP (19). Upon return to upright from the DH position and SHHP, an UBN with torsion lateralized to the involved side may be observed (19). Differentiation between non-ampullary and periampullary obstructions is based on the findings from the head yaw test (the supine recumbent position with the head turned 90° to the ipsilateral (involved) and contralateral (uninvolved) sides). UBN in contralateral yaw and DBN in ipsilateral yaw suggest non-ampullary involvement while DBN in contralateral yaw and UBN in ipsilateral yaw suggest periampullary involvement (19).

Partial or complete obstruction to the lumen of the canal would modify the direction of endolymph displacement and cause a transient high-frequency VOR deficit in the involved vertical canal (36, 37). In individuals with persistent positional DBN, with or without torsion in the DH positions and SHHP and without nystagmus on return to upright, the video head impulse test (vHIT) was able to detect the involved vertical canal of the coplanar pair, either PC-BPPV canal obstruction or AC-BPPV (36, 37). However, in typical acute PC-BPPV, the vHIT was unable to identify the involved canal (38). In this case, the vHIT was not used to identify the involved canal.

In this case, in the SHHP, the 60-s latency before the onset of a transient burst of UBN with left torsion (lateralized to the involved side) suggested an ipsicanal switch from PC-BPPV-cu-la to PC-BPPV-ca-la, implying left PC involvement (14). Computer simulations of the Yarcovino maneuver demonstrate a canal switch from AC-BPPV to PC-BPPV (39). The degree of head extension relative to the earth horizontal in the Yarcovino maneuver and SHHP are similar. A canal switch would result in DBN and possibly a reversal in the direction of torsion. In this case, only UBN with torsion lateralized to the involved side was observed in the SHHP. Therefore, an ipsicanal switch rather than a canal switch occurred. It is hypothesized that the longer latency may reflect the time taken for otolithic debris to move through the ampulla into the long arm of the canal (40), the slow sedimentation velocity caused by small particles (29, 40), silent debris defined as the movement of otolithic debris along the canal wall (29), or neutral head position—an axis of the affected posterior cupula oriented in the earth vertical (22).

Limitations of this case are the need for further positional testing to confirm the left PC-BPPV-cu, neuroimaging to rule out CNS involvement, and vHIT to rule out canal jam or obstruction. Ipsicanal conversion occurred during the SHHP, so no HH or IR was performed. Neuroimaging was not performed. The most common pattern of central positional nystagmus evoked with positional testing is positional DBN (41, 42). When the clinician observes DBN in the provoking position, CNS involvement should be considered. An individual should be referred for a thorough neurologic examination, additional CNS testing, and/or magnetic resonance imaging (MRI) of the brain and posterior fossa if associated auditory or neurological symptoms are present. If symptoms are consistent with BPPV and show no improvement or resolution after two to three sessions of particle repositioning maneuvers (12), a referral should be made for further testing. No associated neurologic signs were not observed in this individual, and the history was not suggestive of CNS involvement. No vHIT was performed to rule out complete or partial occlusion. The location of the obstruction determines the presentation of atypical PC-BPPV, the non-ampullary segment (distal) (18) or the descending periampullary segment (proximal) (19) (Figure 1A). The vHIT has only identified obstructions within the non-ampullary segment of the PC.

The strategy for resolving PC-BPPV-cu is to use the provocative positions that achieve the release of the otolithic debris from the cupula, depending on which side of the cupula the debris is located (10). The SHHP may be used to cause an ipsicanal switch from PC-BPPV-cu to PC-BPPV-ca-la. Once converted to canalithiasis, typical PC-BPPV may be treated with a modified CRP (29) or liberatory maneuver (43). If the debris is located on the side of the cupula near the utricle, the release position can be obtained by having the patient lie on the uninvolved side with the neck flexed 20° and rotated 45° downward (10) (Figure 1E). This is the same position as the side lying position of the CRP, the 180° inverted position of the liberatory maneuver, demi-Semont (lying on the uninvolved side with the neck rotated 45° downward) and 45° forced prolonged positioning (lying on the uninvolved side with the neck rotated 45°downward) (18). If the debris is located on the side of the long arm, a modified CRP or liberatory maneuver may be performed.

## Data availability statement

The raw data supporting the conclusions of this article will be made available by the authors, without undue reservation.

## Ethics statement

The studies involving human participants were reviewed and approved by Institutional Review Board, Midwestern University, Downers Grove, IL, USA. Written informed consent for participation was not required for this study in accordance with the national legislation and the institutional requirements. Written informed consent was obtained from the individual(s) for the publication of any potentially identifiable images or data included in this article.

## Author contributions

JH: conception, study design, acquisition, analysis, interpretation of data, draft of the manuscript, critical reading, and manuscript revision. Author read and approved the submitted version.

## Conflict of interest

The author declares that the research was conducted in the absence of any commercial or financial relationships that could be construed as a potential conflict of interest.

## Publisher's note

All claims expressed in this article are solely those of the authors and do not necessarily represent those of their affiliated organizations, or those of the publisher, the editors and the reviewers. Any product that may be evaluated in this article, or claim that may be made by its manufacturer, is not guaranteed or endorsed by the publisher.

## Supplementary material

Supplementary Video S1 Video, Supplemental Digital Content S1, which illustrates the first variant of the left PC-BPPV—downbeat nystagmus (DBN) in both DH positions and no positional nystagmus upon return to upright from both positions. Upon return to upright, right-beating nystagmus (RBN) is observed. Ipsicanal switch from cupulolithiasis to canalithiasis of the left PC-BPPV during the straight head hang position (SHHP). Digital recordings of ocular nystagmus during positional testing. Digital recordings were edited to reduce the size of files. Digital recordings begin at the point where the individual assumes the provoking position or sitting up except for the SHHP. The SHHP begins at 60 s latency before onset. For each position, digital recordings were reduced from 2 min to 30 s.

Supplementary Video S2 Video, Supplemental Digital Content S2, which illustrates the second variant of the left PC-BPPV—no nystagmus in provoking positions and upbeat nystagmus (UBN) upon return to upright. SHHP were repeated two times. DBN was developed in SHHP. Digital recordings of ocular nystagmus during positional testing. Digital recordings were edited to reduce the size of files. Digital recordings begin at the point where the individual assumes the provoking position or sitting up. For each position, digital recordings were reduced from 2 min to 30 s. Upon sitting up, the individual was provided with light to suppress nystagmus and reduce nausea.
